# Surgery or Consultation: A Population-Based Cohort Study of Use of Orthopaedic Surgeon Services

**DOI:** 10.1371/journal.pone.0065560

**Published:** 2013-06-04

**Authors:** Elizabeth M Badley, Mayilee Canizares, Crystal MacKay, Nizar N. Mahomed, Aileen M. Davis

**Affiliations:** 1 The Arthritis Community Research and Evaluation Unit, Division of Health Care and Outcomes Research, Toronto Western Research Institute, Toronto, Ontario, Canada; 2 Dalla Lana School of Public Health, University of Toronto, Toronto, Ontario, Canada; 3 Institute for Clinical Evaluative Sciences, Toronto, Ontario, Canada; 4 Department of Surgery, University of Toronto, Toronto, Ontario, Canada; 5 Department of Physical Therapy, Rehabilitation Science, University of Toronto, Toronto, Ontario, Canada; 6 Institute of Health Policy, Management and Evaluation, University of Toronto, Toronto, Ontario, Canada; Johns Hopkins Bloomberg School of Public Health, United States of America

## Abstract

**Background:**

This population-based cohort study has the objective to understand the sociodemographic characteristics and health conditions of patients who do not receive surgery within 18 months following an ambulatory visit to an orthopaedic surgeon.

**Methods:**

Administrative healthcare databases in Ontario, Canada were linked to identify all patients making an initial ambulatory visit to orthopaedic surgeons between October 1^st^, 2004 and September 30^th^, 2005. Logistic regression was used to examine predictors of not receiving surgery within 18 months.

**Results:**

Of the 477,945 patients in the cohort 49% visited orthopaedic surgeons for injury, and 24% for arthritis. Overall, 79.3% did not receive surgery within 18 months of the initial visit, which varied somewhat by diagnosis at first visit (84.5% for injury and 73.0% for arthritis) with highest proportions in the 0–24 and 25–44 age groups. The distribution by income quintile of patients visiting was skewed towards higher incomes. Regression analysis for each diagnostic group showed that younger patients were significantly more likely to be non-surgical than those aged 65+ years (age 0–24: OR 3.45 95%CI 3.33–3.57; age 25–44: OR 1.30 95%CI 1.27–1.33). The odds of not getting surgery were significantly higher for women than men for injury and other conditions; the opposite was true for arthritis and bone conditions.

**Conclusion:**

A substantial proportion of referrals were for expert diagnosis or advice on management and treatment. The findings also suggest socioeconomic inequalities in access to orthopaedic care. Further research is needed to investigate whether the high caseload of non-surgical cases affects waiting times to see a surgeon. This paper contributes to the development of evidence-based strategies to streamline access to surgery, and to develop models of care for non-surgical patients to optimize the use of scarce orthopaedic surgeon resources and to enhance the management of musculoskeletal disorders across the care continuum.

## Introduction

Musculoskeletal disorders (MSD) are among the most frequent chronic conditions: each year 22% of the population makes at least one doctor visit for MSD of whom one third see specialists, most frequently orthopaedic surgeons [1], [2]. Access to orthopaedic surgery is a recurring issue particularly in publicly funded healthcare systems such as that of the United Kingdom (UK) or Canada, with concerns both in the demand and supply sides. On the demand side the incidence of MSD including fracture and arthritis are projected to increase as the population ages and obesity increases,[3]–[7] with a concomitant increase in need for surgery, especially for total joint replacement and osteoporosis fracture care [8], [9]. On the supply side there are concerns that typically revolve around pressures caused by the shortage in resources for surgery including orthopaedic surgeons and hospital resources[4], [10]–[13]. There is further concern with system efficiencies[14]–[18] with an emphasis on long wait times for surgery [16], [19].

Our previous study suggested that overall more than two out of three patients seeing an orthopaedic surgeon did not get orthopaedic surgery [20]. This is consistent with reports from the UK [21], [22] and a US study from a capitated population [23]. In principle, seeing a high volume of patients who do not need surgery may put pressure on orthopaedic resources, which may in turn impede access to timely care. Given the expected increase in demand for musculoskeletal care and limited orthopaedic resources, it is important to understand the characteristics of patients who see surgeons but do not need surgery. This study identifies and follows a cohort of patients visiting orthopaedic surgeons in Ontario, Canada. The objective is to understand the sociodemographic characteristics and health conditions of patients who do not receive surgery within 18 months following an ambulatory visit to orthopaedic surgeons.

## Methods

The setting for this study is Ontario, Canada. The target population for this cohort study was the total population of patients who had an ambulatory (office) visit to orthopaedic surgeons between October 1^st^, 2004 and September 30^th^, 2005 (index year) and who had not had surgery within the previous 6 months. They were then followed for 18 months after their initial visit in the index period. The primary outcome was the proportion of patients not receiving surgery, whom we refer to as non-surgical patients.

The study cohort was identified by linking anonymous encrypted health-card data on ambulatory and hospital orthopaedic service utilization from administrative healthcare databases. The Ontario Health Insurance Plan (OHIP) physician’s billing database provided information on orthopaedic ambulatory services, including a diagnostic code, based on a modification of the International Classification of Diseases 9^th^ Edition (ICD-9) [24]. Diagnostic codes in the ambulatory visit database were classified into: *arthritis and related conditions* (osteoarthritis, and other arthritis (e.g. rheumatoid arthritis, traumatic arthritis, synovitis, and ankylosing spondylitis)), *injury and related conditions* (fractures and dislocations, sprains and strains, joint derangement, and other injuries), *bone and joint conditions* (other spine, bone, and unspecified bone joint disorders), and other conditions (including cancers, circulatory diseases, congenital deformities, and conditions of childhood etc) [20]. The Institute of Clinical Evaluative Sciences Physician Database was linked to the OHIP database to identify orthopaedic surgeons. Information on patients’ age, sex and residential postal code was obtained from the Ontario Registered Persons Database. Postal code data were linked to census data for dissemination areas, the smallest geographic unit for which census data are available, and used to estimate neighbourhood income quintiles as a proxy for personal income and hence socioeconomic status (SES) [25]. The Canadian Institute for Health Information Discharge Abstract Database (DAD) and the National Ambulatory Care Reporting System (NACRS) database provided information on inpatient and outpatient surgeries respectively. Orthopaedic surgeries were defined based on the Canadian Classification of Health Intervention (CCI) procedure codes relating to the musculoskeletal system in the hospital databases. Diagnoses in the DAD and NACRS databases associated with surgery were coded according to the International Classification of Diseases 10^th^ Edition (ICD-10) and were allocated to groups similar to those for ambulatory care.

Entry into the cohort was the date of the initial ambulatory visit to an orthopaedic surgeon. As the focus of this study was on ambulatory visits, we excluded cases with likely emergency surgery. Cases were deemed to be an emergency surgery if initial contact with the orthopaedic surgeon was for surgery or if they had surgery within 48 hours of the first ambulatory visit. This time period was chosen as it is the national benchmark for hip fracture surgery [26]. Patients with orthopaedic surgery within 6 months prior to the index visit and therefore likely to be seeing a surgeon for follow-up were also excluded.

### Analyses

Descriptive analyses were conducted to estimate the proportion of patients who did and did not receive an orthopaedic surgery within 18 months of the initial visit to orthopaedic surgeons, by diagnostic groups. Descriptive statistics for demographic and clinical characteristics are presented using frequencies and percentages for categorical variables, and medians for continuous variables. Sensitivity analyses to assess the adequacy of our follow-up time examined if longer follow-up periods would significantly increase the estimated proportion of patients with surgery were carried out by extending the follow-up time to 24 months. Logistic regression was used to assess age, sex, and income quintile as predictors of not receiving surgery for the overall cohort and each diagnostic group.

### Ethics Statement

This study received ethical approval through the institutional research board of Sunnybrook Health Sciences Centre.

## Results

Between October 2004 and September 2005, 521,200 patients accessed orthopaedic services (ambulatory and surgery) in Ontario. There were 477,900 (92.0%) patients who fulfilled the criteria for cohort entry and 43,300 patients were excluded: likely emergency surgery (22,900); surgery within two days of first ambulatory visit (5,200); surgery within the previous six months (15,000). The majority of the exclusions for likely emergency surgery were for injury.

**Figure 1:**
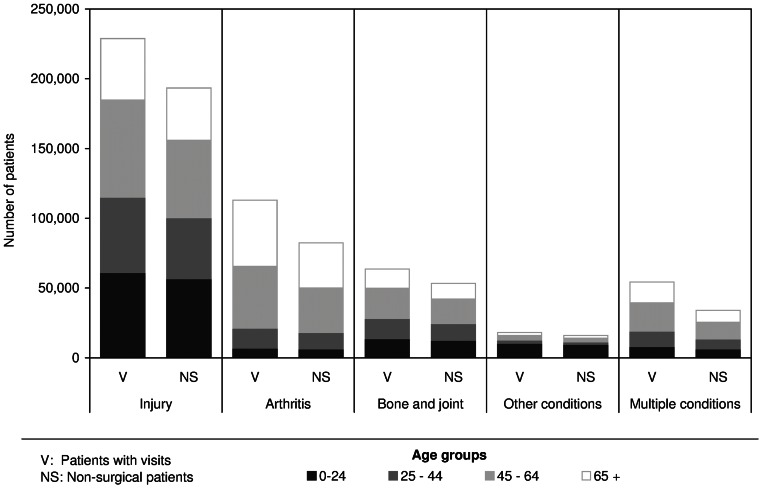
Number of patients with ambulatory visits to orthopaedic surgeons and non-surgical patients by diagnostic groups at initial visit and age groups.

Table 1 shows the characteristics of the cohort, the diagnosis at the initial ambulatory visit to an orthopaedic surgeon, and the proportion of non-surgical patients (patient who did not receive surgery within 18 months of the initial visit to orthopaedic surgeons). Overall, 79.3% of patients were non-surgical, with highest proportions in the 0–24 and 25–44 age groups. Patients aged less than 45 years made up to 40% of all patients with visits, and 45% of non-surgical patients. Almost half of all visits were for injury. Almost a quarter of patients visited for arthritis and related diagnoses, although these patients were mostly aged 45 years or older. The majority of patients visiting for ‘other conditions’ were in the 0–24 age group. Just over 10% of the cohort visited for multiple conditions. The distribution of patients with an ambulatory visit by income quintile was skewed towards higher incomes, but the opposite relationship was seen for non-surgical patients. The proportion of non-surgical patients varied somewhat by diagnostic group (Figure 1), and was highest for injury and particularly fractures (94%). This is not unexpected as we excluded likely emergency surgery from the cohort. The proportion of non-surgical patients was lowest for multiple diagnoses (63%) and osteoarthritis (65%). The latter was likely consultation for consideration of joint replacement surgery.

**Table 1:** Characteristics of the cohort groups at initial ambulatory visit with orthopaedic surgeons (October 2004– September 2005), and proportion receiving non-surgical care within 18 months of initial visit.

	With office visits		Receiving non-surgical care	
	Number	%	Number	%
**All**	**477,945**	**100.0**	**379,147**	**79.3**
**Sex**				
Female	248,788	52.1	195,568	78.6
Male	229,157	47.9	181,324	79.1
**Age at initial consult (years)**				
0–24	100,014	20.9	91,122	91.1
25–44	96,406	20.2	76,735	79.6
45–64	158,905	33.2	119,532	75.2
65+	122,620	25.7	91,758	74.8
**Income quintile**				
Quintile 1 (lowest)	81,510	17.1	65,441	80.3
Quintile 2	88,396	18.5	69,836	79.0
Quintile 3	92,559	19.4	73,050	78.9
Quintile 4	99,576	20.8	78,460	78.8
Quintile 5 (highest)	105,436	22.1	82,216	78.0
Missing	10,468	2.2	10,144	96.9
**Diagnostic groups**				
One condition	423,625	88.6	345,088	81.5
a) Injury	228,825	47.9	193,398	84.5
Fractures & dislocations	90,764	19.0	85,227	93.9
Sprains & strains	86,800	18.2	70,916	81.7
Joint derangement	43,745	9.2	30,709	70.2
Other injuries	7,516	1.6	6,546	87.1
b) Arthritis	112,997	23.6	82,474	73.0
Osteoarthritis	66,471	13.9	43,206	65.0
Other arthritis	46,526	9.7	39,268	84.4
c) Bone and joint	63,620	13.3	53,289	83.8
Other and unspecified bone and joint	27,746	5.8	22,890	82.5
Spine	25,036	5.2	22,107	88.3
Bone	10,838	2.3	8,292	76.5
d) Other conditions^1^	18,183	3.8	15,927	87.6
Multiple conditions^2^	54,320	11.4	34,059	62.7

1. Includes cancer, circulatory diseases, and conditions of the childhood. ^2.^ Range 2–7.

**Figure 2:**
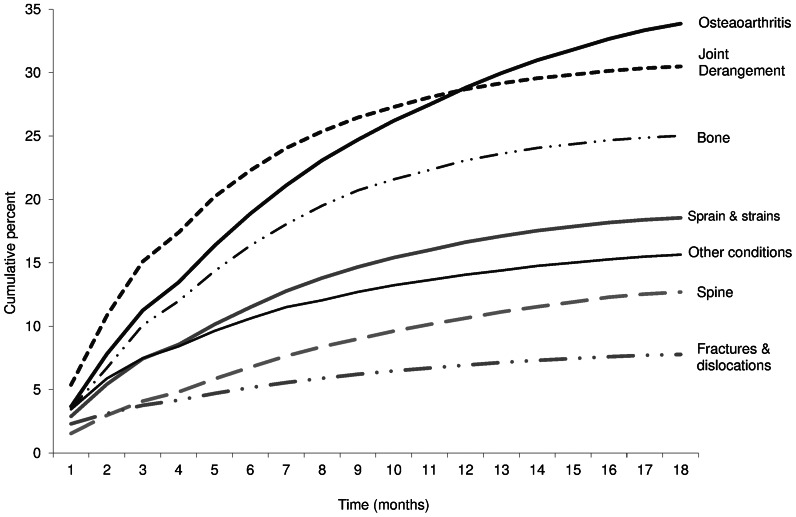
Cumulative percent curve for proportion of patients receiving orthopaedic surgery by diagnostic groups at initial ambulatory visit to orthopaedic surgeons.

Figure 2 shows the cumulative proportion of persons having orthopaedic surgery up to 18 months of the initial ambulatory visit in the index year by diagnostic group. After 18 months the curve of the trajectory had flattened suggesting that this time period had captured the majority of the surgeries. Sensitivity analyses showed that longer follow-up periods would not substantially increase the estimated proportion of patients with surgery. For example, at 18 months the proportion of patients with visits for osteoarthritis who had surgery was 35% and the projected proportion at 24 months was 36.9%.

Regression analysis for each diagnostic group showed that younger patients were less likely to have surgery than those aged 65+ years (Table 2). For injury and ‘other conditions’ the odds of not-getting surgery were significantly higher for women than men; while the opposite was true for arthritis and bone conditions. The odds of not getting surgery were greater for patients in the lowest income quintile compared to the highest for injury, arthritis, and bone conditions.

**Table 2:** Predictors of receiving non-surgical care by diagnostic groups at the initial ambulatory visit to orthopaedic surgeons: findings from multiple logistic regression.

Diagnostic group at initial visit	Sex		Age groups (Ref age 65+)						Income quintile	
	Female vs Male		0–24		25–44		45–64		Q1 (lowest) vs Q5 (highest)	
	OR	95% CI	OR	95% CI	OR	95% CI	OR	95% CI	OR	95% CI
*One condition*	*1.09*	*1.08*–*1.10*	*3.57*	*3.45*–*3.70*	*1.30*	*1.27*–*1.33*	*1.02*	*1.00*–*1.04*	*1.20*	*1.18*–*1.23*
a) Injury	1.25	1.23–1.27	2.38	2.33–2.44	0.79	0.76–0.81	0.72	0.70–0.75	1.32	1.27–1.37
Fractures & dislocations	1.33	1.27–1.41	2.70	2.44–3.03	0.74	0.68–0.80	0.65	0.61–0.70	1.79	1.67–1.92
Sprains & strains	1.10	1.06–1.14	2.17	2.04–2.33	1.10	1.05–1.15	0.96	0.92–1.01	1.08	1.01–1.15
Joint derangement	1.30	1.23–1.37	1.05	0.97–1.15	0.75	0.70–0.80	0.75	0.70–0.81	1.20	1.12–1.30
Other injuries	1.20	1.04–1.43	1.75	1.35–2.50	0.58	0.47–0.76	0.55	0.45–0.71	1.35	1.09–1.79
b) Arthritis	0.96	0.93–0.99	5.26	5.00–5.56	2.08	2.00–2.17	1.19	1.16–1.22	1.09	1.05–1.12
Osteoarthritis	0.97	0.94–1.00	3.23	2.44–4.76	1.67	1.56–1.79	1.02	0.98–1.06	1.12	1.06–1.19
Other arthritis	0.90	0.86–0.94	2.50	2.27–2.78	1.12	1.04–1.22	0.88	0.83–0.94	0.95	0.88–1.04
c) Bone and joint	0.88	0.84–0.92	2.33	2.17–2.50	1.00	0.94–1.06	0.90	0.85–0.95	1.19	1.11–1.28
Unspecified bone and joint disorders	1.12	1.05–1.20	1.82	1.64–2.04	0.88	0.80–0.97	0.81	0.75–0.88	1.18	1.05–1.33
Spine	0.96	0.88–1.05	2.63	2.27–3.13	1.22	1.10–1.37	1.32	1.19–1.47	1.11	0.98–1.28
Bone	0.58	0.52–0.65	3.03	2.56–3.70	0.88	0.76–1.05	0.76	0.68–0.87	1.03	0.88–1.23
d) Other conditions	1.06	0.97–1.18	1.85	1.61–2.17	0.85	0.73–1.03	0.74	0.64–0.88	0.88	0.76–1.04
*Multiple conditions*	*1.05*	*1.02*–*1.09*	*2.22*	*2.08*–*2.38*	*1.22*	*1.16*–*1.28*	*0.97*	*0.93*–*1.02*	*1.14*	*1.08*–*1.20*
*All conditions*	*1.08*	*1.06*–*1.09*	*3.45*	*3.33*–*3.57*	*1.30*	*1.27*–*1.33*	*1.01*	*0.99*–*1.03*	*1.19*	*1.16*–*1.22*

## Discussion

This is the first study that we are aware of that has looked in detail at the characteristics of patients who do not get surgery following an ambulatory visit to an orthopaedic surgeon. We showed that four out of five patients visiting orthopaedic surgeons in Ontario, Canada did not receive surgery when followed up to 18 months after their initial ambulatory visit. In presenting these findings we do not mean to imply that patients who needed surgery did not receive it. The shape of the trajectory of time to surgery (Figure 2) suggests that all patients needing surgery received surgery, albeit after a wait time particularly for patients with OA. On the contrary, the high proportion of non-surgical patients across all diagnostic groups highlights the important role that orthopaedic surgeons have in providing specialist input for diagnosis and advice on management of patients with musculoskeletal injury, arthritis and other conditions.

Our findings raise several questions which are discussed below. Firstly, does the high volume of non-surgical patients increase waiting times for a first appointment with an orthopaedic surgeon and thus impede access for individuals in urgent need of surgical care? Secondly, given the high volume of non-surgical patients, does everyone seeing an orthopaedic surgeon need the surgeon’s special skills and could some be seen by others? Thirdly, related to this, is the extent to which surgeons’ training, which focuses on surgical skills, adequately prepares them for the non-surgical management of MSD patients.

In many jurisdictions including Canada, there has been concern about wait times, and specifically on the time elapsed between the decision for surgery and the intervention with particular emphasis on wait times for total joint replacement surgery[27]–[29]. Less emphasis has been placed on wait time from referral to orthopaedic consultation, which had been identified as a barrier to timely access to orthopaedic care [30], [31]. However, according to a report by a Canadian organization concerned with public policy the waiting time for an appointment to see an orthopaedic surgeon is similar in magnitude to the wait time between the decision for surgery and surgery [32]. The findings from this study focuses attention on the wait time between referral and appointment with the surgeon, the so-called ‘wait 1′, sets discussion of wait times in the larger context of the competing priorities of all patients seen.

In response to concerns about wait times for orthopaedic surgery and increasing demand, a diversity of alternative models of care mainly located in hospital settings have been developed especially in countries with publicly funded healthcare systems [15], [16], [18], [33].These models vary somewhat by type of health care system. In fee-for-service settings, such as Canada, most models have been developed in response to concerns about wait times for joint replacement surgery, and have been fuelled by financial incentives. They are targeted to ‘triage’ patients to surgery, particularly those presenting for consideration of joint replacement surgery[34]–[36]. Relatively less attention has been paid to the needs for conservative management for those patients deemed ineligible for surgery. Models to triage patients referred with non-arthritis conditions or for consideration for surgery other than total joint replacement are only just beginning to be developed. As this study shows, only a minority of both surgical and non-surgical patients seen by orthopaedic surgeons have arthritis, and less than 25% of all surgeries are joint replacement surgeries [20]. Furthermore, how and whether wait times to see a surgeon for consideration of total joint replacement surgery might be affected by the total spectrum of conditions presenting to orthopaedic surgeons has received relatively little attention.

A somewhat different approach has been possible in health care systems such as that of Australia and particularly the UK where national standards are set by the Department of Health [37] and where there is funding for salaried health care professionals. Here a more comprehensive approach has been taken to reducing wait times and improve management of MSD in general. These approaches include models of care which use multi-disciplinary teams as gatekeepers to orthopaedic care, [21], [38]–[44] dealing with the full spectrum of patients referred with MSD. Such teams frequently include physiotherapists working in extended practice/roles and may also include primary care physicians and other health professions. Evaluation of these models has shown them to be acceptable to patients, [38], [41] and to have reduced wait times from referral to first appointment, [41], [45] lower costs [45] and an increased surgical conversion rate for those patients who were triaged to surgeons [21], [42]. In cases where the teams are located at the intersection of primary and secondary care a further benefit has been reduced referrals to secondary care with many patients being treated with conservative management [42]. Evaluation of the performance of extended scope physiotherapists in such roles have good agreement with surgeons about diagnosis and/or need for surgery[45]–[47].

The experience outlined above suggests that a proportion of patients referred to orthopaedic surgeons could be seen by others, particularly those needing conservative management. It is likely that most of the patients seen were referrals by primary care physicians. Studies show that primary care physicians lack training in the management of arthritis and other MSD [48], [49]. Their need for support is reflected by the fact that one third of all doctors visits for MSD are to specialists [2]. A review from the UK on the quality of general practitioner diagnosis and referral [50] indicated that a high proportion of referrals to orthopaedic surgeons were assessed by specialists to be unnecessary, more appropriate for rheumatology, [43] and that about half of referrals could have been treated in community settings [51]. How support for the non-surgical management of MSD can be provided and by whom is an issue that needs to be grappled with as part of health care and primary health care reform in Canada and elsewhere. Increasing demand with the aging of the population and need for cost containment is creating pressures for the transfer of clinical care from hospital to primary care settings [52]. The findings from this paper provide a background for discussion of models of care possibly targeted to specific sub-populations, such as the younger population who are less likely to need surgery or need care for injuries. Such models could perhaps take advantage of existing expertise, like sports medicine and physical therapy, to provide specialist advice at the primary care level. Therefore facilitating timely access to advice and conservative management of patients with MSD and potentially averting some referrals to orthopaedic surgeons which in turn reduce pressure on wait times for orthopaedic consultation.

How best to provide specialist input into the conservative management of MSD also raised the question of the extent to which a surgeon’s training prepares them for this role. There have been shown to be deficiencies in the physical examination knowledge and skills of orthopaedic residents [53]. A recent cohort study of patients with osteoarthritis referred to surgeons showed more than half were not provided with information on osteoarthritis, pain management or exercise [54]. Despite the high proportion of non-surgical referrals, the role of orthopaedic surgeons in the medical care of MSD and the training of surgeons for the provision of non-surgical care is not widely recognized [55], [56].

The findings from this study also point to continued inequities in access to surgery: individuals of low SES were less likely to visit surgeons and were more likely to be non-surgical, as were women. The gender and SES differences found in this study are in line with previous studies on inequities in access to surgery[8], [57]–[65]. SES differences could reflect a variety of issues including patients’ ability to navigate the system as well as availability of services, particularly in rural or low income areas. Such differences ideally need to be factored into the further development of models of care.

This study, like all studies which use administrative data, has several limitations; we have details of neither the reasons for referral nor the factors influencing the surgeons’ decisions. Also we have no information about what proportion were referrals from other than primary care physicians, came via the emergency room, or where follow-up visits by patients with surgeries more than six months earlier. To create the cohort several somewhat arbitrary assumptions were made; we excluded patients with likely emergency surgery and those whose visits were assumed to be follow-up visits from surgery in the previous six months. However, the number of patients excluded was relatively small. Linking surgeries to the physician billings database is not straightforward. The ambulatory and hospital care databases used different classifications of diagnosis (a sub-set of ICD-9 codes for ambulatory visits data and ICD-10 for data on hospitalizations), and therefore the codes used in the two databases may not be equivalent making it difficult to directly attribute the surgery to a previous visit. This could lead to errors in our estimate of the proportion in each diagnostic group having surgery and also means that we cannot estimate wait times.

A strength of this study is that it captures all ambulatory visits to orthopaedic surgeons in the total population of the largest province in Canada. We do not think that high proportion of non-surgical patients indicates that patients who needed surgery were not getting it. Our sensitivity analysis of time to surgery (Figure 2) showed it is unlikely that a longer follow-up period would have substantially changed these results. The high proportion of non-surgical patients is also compatible with estimates that can be derived from a paper presenting data on a capitated population in the US, [23] and two UK reports [21], [22]. It is also compatible with surveys of Ontario Orthopaedic Surgeons that showed half of a surgeons’ time was spent in office-based care [10].

### Conclusions

This paper provides evidence to contribute to a debate on resource allocation and delivery of services to meet the needs of patients with musculoskeletal disorders. Four out of five patients did not receive surgery following an orthopaedic surgeon visit. While it is likely that all patients needing surgery received it, further investigation is needed to determine whether the high caseload of non-surgical cases affects waiting times for appointments to see a surgeon, and is thus a barrier for access to timely surgical intervention for those in urgent need. An implication of our findings is that there may be a need to develop alternative strategies to provide ‘specialist’ input for non-surgical musculoskeletal care, particularly with the anticipated increasing demands for surgery as the population ages. Such models could also contribute to optimizing the use of orthopaedic surgeon time, and at the same time enhance the management of musculoskeletal disorders in primary care. Many of the non-surgical patients had injury and/or were more likely to be younger, suggesting the possibility of targeted models of care. This paper provides the beginning of an evidence-base from which to develop strategies to streamline access to care for those who need surgery, and to develop interventions to meet the needs of those who do not.
